# Disease-Associated Dopamine Receptor D2 Variants Exhibit Functional Consequences Depending on Different Heterotrimeric G-Protein Subunit Combinations

**DOI:** 10.3390/biomedicines13010046

**Published:** 2024-12-28

**Authors:** Nele Niebrügge, Olga Trovato, Roman Praschberger, Andreas Lieb

**Affiliations:** 1 Institute of Pharmacology, Medical University of Innsbruck, 6020 Innsbruck, Austria; 2 Institute of Human Genetic, Medical University of Innsbruck, 6020 Innsbruck, Austria

**Keywords:** GPCRs, dopamine receptors, movement disorders

## Abstract

**Background:** Dopamine receptors (DRs) are G-protein-coupled receptors (GPCRs) found in the central nervous system (CNS). DRs are essential for mediating various downstream signaling cascades and play a critical role in regulating the dopaminergic nigrostriatal pathway, which is involved in motor control. Recently, mutations in DRD2 (WT), p.Ile212Phe (I212F), and p.Met345Arg (M345R) have been associated with hyperkinetic movement disorders and shown to alter heterotrimeric G-protein complex signaling and β-arrestin recruitment. **Methods**: To conduct a detailed investigation of the I212F and M345R functional phenotypes, we used the TRansdUcer PATHway (TRUPATH) assay to study heterotrimeric G-protein recruitment and the Parallel Receptorome Expression and Screening via Transcriptional Output (PRESTO-Tango) assay to evaluate transcriptional activation following arrestin translocation for β-arrestin recruitment. **Results:** In our study, we could confirm the reported mutant’s loss-of-function phenotype in β-arrestin 2 recruitment (reduced agonist potency and decreased maximal signaling efficacy in comparison to the WT). However, a detailed analysis of basal/constitutive activity also revealed a gain-of-function phenotype for mutant M345R. For a more comprehensive investigation of heterotrimeric G-protein complex signaling, we investigated the impact of WT mutants in combination with (i) a specifically suggested assay, and (ii) the most abundantly expressed heterotrimeric G-protein complex combinations in WT receptor-enriched regions. We were able to confirm the reported gain-of-function phenotype by Rodriguez-Contreras et al. and extend it by the use of the most abundant heterotrimeric G-protein subunits, Gα_oA_ and Gα_i1_, β_1_ and β_2_, and γ_3_ and γ_7_, in mouse and human basal ganglia. **Conclusions:** Although our results indicate that the interaction of the two variants with the most highly expressed heterotrimeric G-protein complex subunit combinations also results in a gain-of-function phenotype, they also clearly demonstrate that the phenotype can be significantly altered, dependent on heterotrimeric G-protein complex expression.

## 1. Introduction

Dopamine receptors (DRs) belong to the protein superfamily of cell membrane receptors known as G-protein-coupled receptors (GPCRs) [1]. GPCRs represent the most targeted drug class of Federal Drug Administration (FDA)- and European Medicine Agency (EMA)-licensed drugs [2,3]. Expressed in the central nervous system (CNS), DRs are involved in the modulation of important physiological functions such as the reward system, motor control, cognition, memory, emotion, and learning [4,5]. DRs are classified into two main groups based on their G-protein-dependent signaling pathways: the D1-like receptors (D1 and D5), which primarily couple to G_s/olf_ proteins and promote cellular excitation, and the D2-like receptors (D2, D3, and D4), which signal via G_i/o_ proteins to induce cellular inhibition. Activation of GPCRs leads to interaction with G-proteins, which are heterotrimeric complexes composed of three different subunits: Gα-, Gβ-, and Gγ-. Gα subunits are typically classified into four groups (Gα_s_, Gα_i/o_, Gα_q/11_, and Gα_12/13_), depending on the Gα subunit’s sequence similarities and downstream signaling [6]. Following agonist binding and GPCR activation, the Gα subunit dissociates from the Gβ/γ complex and both independently interact with downstream effector proteins, including adenylyl cyclase, phospholipase C, cGMP phosphodiesterases, and Rho guanine nucleotide exchange factors (RhoGEFs) [7,8]. The downstream signaling effects are predominantly determined by the Gα subunit; however, the Gβ/γ complex also interacts with different downstream molecules such as G-protein-coupled inwardly rectifying potassium channels (GIRKs), thereby modulating the cellular response [9,10,11]. In humans, multiple Gα-, Gβ-, and Gγ-subunits have been identified [12,13,14], where each of them is differentially expressed in the human brain [15] and exists in multiple isoforms, providing a wide range of potential heterotrimeric G-protein combinations. These combinations are essential for activating downstream signaling pathways, thereby specifically modulating various physiological processes in response to extracellular stimuli. G-protein-mediated signaling is terminated by kinases called GPCR kinases (GRKs) [16,17]. These kinases phosphorylate specific amino acids within the GPCR intracellular domain, which leads to β-arrestin recruitment and receptor internalization and also promotes β-arrestin-mediated signaling [18,19,20]. The interaction between GPCRs and β-arrestins prevents G-protein coupling, thereby further regulating both the intensity and duration of the cellular response [21]. This adaptive nature of GPCR signaling is crucial for maintaining cellular responsiveness and homeostasis [22,23]. The β-arrestin family comprises four subtypes, including visual arrestin 1, arrestin 2 (β-arrestin 1), arrestin 3 (β-arrestin 2), and visual arrestin 4 [24]. β-arrestin 1 and β-arrestin 2 are both expressed in the brain and share similar sequences and functions. These proteins are critical for the desensitization, internalization, and signaling of GPCRs. Further, they have also been implicated in the pathogenesis of neurodegenerative diseases, including Parkinson’s disease, where β-arrestins are elevated, Alzheimer’s disease, where they facilitate the production and accumulation of β-amyloid, and frontotemporal dementia [25].

In the human brain, D2 is highly expressed in striatal spiny projection neurons and therefore forms an integral part of the indirect pathway of the cortico-basal ganglia-thalamo-cortical (CBGTC) neural circuit, which is essential for motor control [26,27]. These neurons are preferentially vulnerable in Huntington’s disease [28], where their loss is associated with the typical hyperkinetic movement disorder symptom termed chorea. The loss of dopaminergic neurons disrupts this network, leading to dysfunction of the basal ganglia circuit and the subsequent movement deficit symptoms observed in different human neurological disorders [29]. The observed chorea motor symptoms are often accompanied by psychiatric symptoms, such as depression, anxiety, and bipolar disorders [30]. Given D2’s essential role in extrapyramidal motor control, it is a well-established target for drugs used to treat movement disorders [31,32]. Recent studies have linked mutations in the intracellular cytoplasmic loop (ICL3) of the D2 (WT) to an autosomal dominant genetic disorder characterized by chorea and dystonia [32,33,34]. These include the single-point mutations isoleucine (I) to phenylalanine (F) at position 212 and methionine (M) to arginine (R) at position 345 (M345R), of which M345R appears to result in a more severe phenotype based on the clinical information of two affected individuals that are thus far described [33]. Rodriguez-Contreras et al. [32,35] have already characterized the Gα_i/o_ signaling (Gα_oA_ and Gα_i2_ signaling for quinpirole and dopamine, and Gα_i1_, Gα_i3_, Gα_oB_, and Gα_z_ for quinpirole in combination with β_1_ and γ_2_) of the pathogenic mutations and their effects on β-arrestin 2 recruitment. However, the screening of the downstream signaling pathways remains incomplete. Therefore, additional studies are required to fully elucidate WT mutant modifications in β-arrestin and heterotrimeric G-protein complex interactions. Using the native ligand dopamine and the agonist pramipexole, we extend the known phenotype by integrating basal (i.e., constitutive) β-arrestin 2 signaling and additional heterotrimeric G-protein subunits, considering that: (i) Gα coupling is also dependent on β/γ subunit combinations, and (ii) the levels of G-protein subunits vary across different brain regions. Our study therefore investigates WT and mutant G_i/o_-dependent signaling using the most abundantly expressed heterotrimeric G-protein subunits, Gα_oA_ and α_i1_, β_1_ and β_2_, and γ_3_ and γ_7_, in mouse and human basal ganglia, a region implicated in the manifestation of most chorea symptoms.

## 2. Materials and Methods

### 2.1. Chemicals

Chemical reagents used in experiments were obtained from the following institutions: Fetal bovine serum (FBS), GlutaMAX™ supplement, OptiMEM reduced serum medium phenolred-free, Penicillin/Streptomycin (P/S), Dulbecco’s Modified Eagle Medium (DMEM) phenolred-free, Dulbecco’s modified eagle medium (DMEM) + phenolred and GlutaMAX™, Trypsin-EDTA, and Puromycin from Gibco (Waltham, MA, USA). Ampicillin sodium salt, Calcium chloride, D-Glucose, Dimethyl Sulfoxide, Glycerol (86%), HEPES, LB agar, Magnesium Chloride Hexahydrate, Miller’s LB broth base, Sodium chloride, and Tris from Carl Roth (Karlsruhe, Germany). dNTPs (10 nM), Dnpl Enzyme, Q5 High GC Enhancer (5X), Q5 High-fidelity DNA Polymerase, Q5 reaction buffer (5X), rCutSmart^®^ buffer, Gel loading dye (purple (6X)), and DNA Ladder (1 kb plus) from New England Biolabs GmbH (Ipswich, MA, USA). Magnesium sulfate heptahydrate, Potassium chloride, Potassium dihydrogen phosphate, and Sodium dihydrogen phosphate from Merck (Darmstadt, Germany). Hygromycin B, Phosphate Saline Buffer (PBS), and Agarose from Thermo Fisher Scientific bioreagents (Waltham, MA, USA). Coelenterazine 400a and D-Luciferin firefly type from NanoLight (Norman, OK, USA) and Polyethylenimine (PEI) MAX from Polysciences, Inc. (Warrington, PA, USA). Dopamine hydrochloride from Sigma Aldrich (St. Louis, MO, USA). Pramipexole dihydrochloride monohydrate from TCI (Taipei, Taiwan).

### 2.2. Molecular Biology

GFP-DRD2 was a gift from Jean-Michel Arrang (Addgene plasmid #24099, http://n2t.net/addgene:24099, accessed on 25 December 2024; RRID: Addgene_24099) [36]. The mutations were introduced into GFP-DRD2 with IVA cloning [37]. The primers used for D2 mutagenesis of I212F are 5’- GCTGGTCTACATCAAGTTCTACATTGTCCTCCGCAGAC-3′ and: 5′-GAACTTGATGTAGACCAGCAGGG-3′ for the mutant, and 5′-AAGAAAGCCACTCAGAGGCTCGCCATTGTTCTCGGC-3′ and 5′-CCTCTGAGTGGCTTTCTTCTCCT-3′ for M345R. For the PRESTO-Tango assay, DRD2-Tango was used, a gift from Bryan Roth (Addgene plasmid #66269; http://n2t.net/addgene:66269, accessed on 25 December 2024; RRID:Addgene_66269) [38]. Please note that the coding, not codon-optimized, sequence of GFP-DRD2 was inserted into DRD2-Tango using IVA cloning. Mutants in DRD2-Tango were also introduced by site-directed mutagenesis with the same primers used for mutagenesis in GFP-DRD2. The G-protein subunits were obtained from Addgene. Gα_i1_-RLuc8 (Addgene, #140973), Gα_i2_-RLuc8 (Addgene, #140974), Gα_i3_-RLuc8 (Addgene, #140975), Gα_oA_-RLuc8 (Addgene, #140976), Gα_oB_-RLuc8 (Addgene, #140977), Gβ_1_ (Addgene, #140987), Gβ_2_ (Addgene, #166772), Gβ_3_ (Addgene, #140988), Gγ_3_-GFP2 (Addgene, #166775), Gγ_7_-GFP2 (Addgene, #166778), Gγ_8_-GFP2 (Addgene, #140990), and Gγ_9_-GFP2 (Addgene, #140991) were gifts from Bryan Roth (Addgene kit #1000000163). All constructs were verified by sequencing reactions by Eurofins Genomics (Ebersberg, Germany) or Microsynth (Balgach, Switzerland).

### 2.3. Cell Culture and Transfection

HEK293 cells, a gift from Prof. Joerg Striessnig (University of Innsbruck, Austria), or HTLA cells, stably expressing a tetracycline transactivator domain (tTA)-dependent luciferase reporter gene and a human β-arrestin 2–Tobacco Etch Virus (TEV) fusion gene (a gift from Dr. Richard Axel) [39] were used for all experiments. Cells were maintained at 37 °C and 5% CO_2_ (Heracell™ 150i) in Dulbecco’s modified eagle medium (DMEM) + GlutaMAX™ (#10567014), with 10% fetal bovine serum (FBS; #A5256701), 1% Penicillin/Streptomycin (P/S #11548876), 2 µg/mL Puromycin (A11138-03), and 100 µg/mL Hygromycin B for HTLA. For transfection, ~900,000 cells/well were seeded onto a 6-well culture dish (#353224, Falcon). All plasmids (each 1 µg) were added to OptiMEM (#10149832) and mixed with the polycationic transfection agent polyethylenimine (PEI) MAX (#24765). The mixture was added dropwise to the cells after 10 min of incubation at room temperature. For experiments, the cells were harvested 24 h after transfection and ~30,000 cells/well were seeded onto a 96-well plate (#655083; Greiner) with DMEM without phenolred (#21063045), supplemented with 1% P/S, 1% GlutaMAX™ (#35050-061), and 1% sodium pyruvate (#11360-070), and incubated at 37 °C and 5% CO_2_ for 24 h.

### 2.4. G-Protein Signaling Assay

To measure the G-protein interaction and constitutive activity of the WT, I212F, and M345R, the TRansdUcer PATHway (TRUPATH) assay was used as published by Olsen et al. [40]. RLuc8-tagged Gα proteins and GFP2-tagged Gγ proteins were used in a ratio of 1:1:1:1 of D2 receptor/α-/β-/γ-subunits. For measurement of Bioluminescence Resonance Energy Transfer (BRET), the medium in the 96-well plates was removed and replaced with 70 µL assay buffer (1× Hank’s balanced salt solution (HBSS) plus 20 mM HEPES pH 7.4 (#HN78.2) and Coelenterazine 400a (final conc. 35 µg/mL; #340-5) with a multipipette. After 5 min of incubation at 30 °C, BRET was measured using a Spark^®^ Tecan plate reader with emissions of 260–440 nm (RLuc8-coelenterazine 400a) and 505–575 nm (GFP2) (integration times of 1 s per well). Then, 20 mM Dopamine hydrochloride in assay buffer (#H8502-5G) was prepared fresh, diluted to final concentrations of 333 µM to 0.333 nM in assay buffer, and 30 µL of the dilutions were added as given to achieve a total volume of 100 µL with a final concentration between 100 µM and 0.1 nM. After an additional incubation period of 10 min at 30 °C, another BRET measurement was performed.

### 2.5. β-Arrestin 2 Signaling Assay

For β-arrestin 2 signaling, the Parallel Receptorome Expression and Screening via Transcriptional Output—TANGO (PRESTO-Tango) assay with transcriptional activation following arrestin translocation was used as published in Kroeze et al. [39]. HTLA cells were transfected with 1 µg of Tango-plasmid and 1 µg mRuby2, a gift from Michael Lin (Addgene plasmid #40260; http://n2t.net/addgene:40260, accessed on 25 December 2024; RRID: Addgene_40260) [41]. Following splitting into 96-well plates, cells were treated with Pramipexole dihydrochloride monohydrate (#P2073-100MG), freshly prepared and diluted to final concentrations between 0.3 µM to 0.01 nM in a total volume of 100 µL in DMEM without phenolred supplemented with 1% P/S, 1% Glutamax, 1% sodium pyruvate, 100 µg/mL Hygromycin B (#J60681), and 2 µg/mL Puromycin (#A11138-03). Dopamine could not be used in this assay as it is an unstable compound and therefore not suitable for overnight cell treatments.

Following 18–24 h of incubation at 37 °C and 5% CO_2_, the cell culture medium was removed and D-Luciferin (final conc. 0.5 mg/mL; #306) was added to the assay buffer with a multipipette. mRuby2 fluorescence (excitation: 540–560 nm, emission: 580–600 nm) and luminescence (integration time 1 s) were recorded at 30 °C with a Spark^®^ Tecan plate reader.

### 2.6. Data Analysis and Statistics

For analysis, GraphPad Prism 8.0.2 (GraphPad Software, Boston, MA, USA) was used. For the β-arrestin 2 recruitment assay, relative luminescence units (RLU) were normalized to mRuby2 as the transfection control and to the mean of the maximum RLU of the WT same-day control. For the TRUPATH assay, netBRET (GFP2/RLuc8) was normalized to the mean basal values (vehicle treatment) of the WT same-day control. Subsequently, all values were normalized to the mean of the maximum G-protein activation of the WT same-day control. Concentration–response curves were generated from normalized data and plotted with a Log 10 scale. A non-linear regression fit was performed. Basal activity (constitutive activity) was compared using bottom values (mean ± SEM) from individual non-linear regression fits, indicating receptor activity without agonist application. Maximal efficacy (E_max_) was evaluated using Top values (mean ± SEM) of individual concentration–response fits, indicating a maximal receptor response to agonist application. In all the experiments, I212F and M345R were compared to WT (one-way ANOVA with Dunnett’s post hoc test, or Student’s *t*-test, as indicated). Data are shown as mean ± SEM, n/N technical/biological replicates.

## 3. Results

### 3.1. β-Arrestin 2 Recruitment of the WT, I212F, and M345R

**Figure 1:**
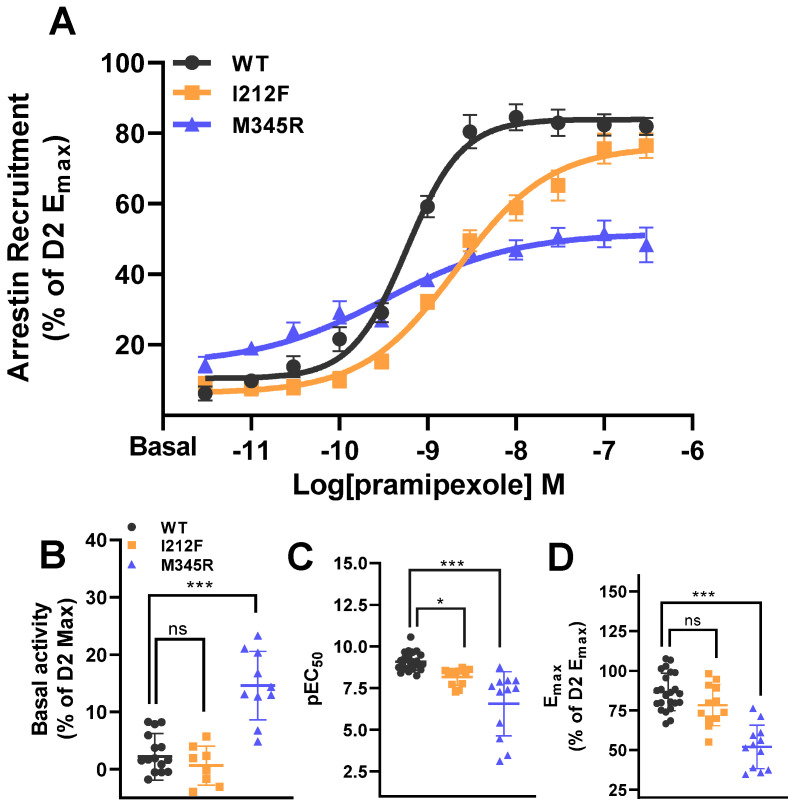
β-arrestin 2 recruitment of the WT, I212F, and M345R upon pramipexole application. (**A**) For concentration–response curves, RLU was normalized to the mean of the max pramipexole response (E_max_) of the WT same-day control (Arrestin Recruitment (% of WT E_max_)). (**B**) Constitutive activity of I212F and M345R compared to the WT, shown as a percentage of the mean WT E_max_ same-day control (Basal activity (% of WT Max)). (**C**) pEC_50_ values for the WT, I212F, and M345R are indicated. (**D**) E_max_ of the WT, I212F, and M345R, normalized to the mean of the maximum pramipexole response of the WT same-day control (E_max_ (% of WT E_max_)). Statistical analysis was conducted using one-way ANOVA with Dunnett’s post hoc test as given in Table 1, indicated as: * *p* < 0.05; *** *p* < 0.001; ns—not significant. Data shown as mean ± SEM.

The PRESTO-Tango assay was used, as published by Kroeze et al. [39], to determine β-arrestin 2 recruitment following pramipexole-dependent receptor activation (Figure 1A).

We could identify a significantly increased constitutive activity for M345R (Figure 1B; Table 1). Consistent with Rodriguez-Contreras et al., we could observe a decreased sensitivity of mutants to agonists, using pramipexole (Figure 1C; Table 1). We could also confirm the significant decrease in β-arrestin 2 recruitment efficacy (E_max_) for M345R but not for I212F compared to the WT following pramipexole application (Figure 1D; Table 1).

### 3.2. WT, I212F, and M345R G-Protein Activation

**Figure 2:**
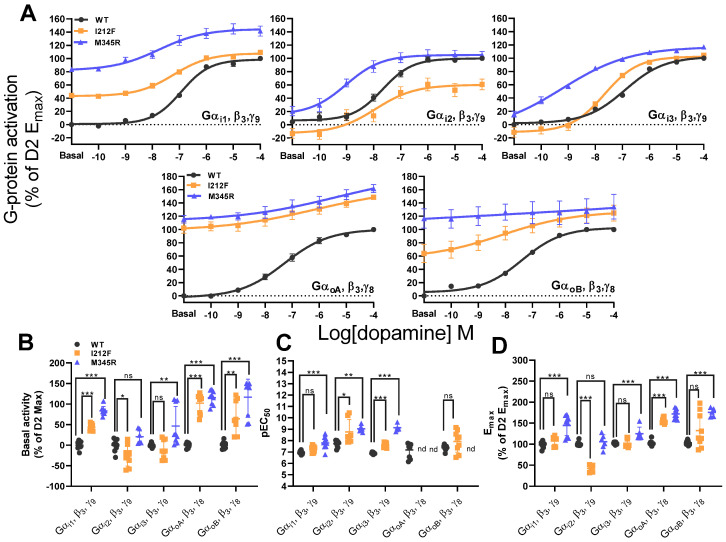
G-protein activation of the WT, I212F, and M345R. (**A**) Concentration–response curves for the WT, I212F, and M345R with Gα_i/o_, β-, and γ-subunits as indicated, following dopamine application. netBRET (GFP2/Rluc8) values are normalized to the mean of the max dopamine response of the WT same-day control and reported as a percentage of the WT same-day control (% of G-protein activation (WT E_max_)). (**B**) Basal activity for the WT, I212F, and M345R are plotted as percentages of the mean WT E_max_ same-day control (Basal activity (% of WT Max)). (**C**) Comparison of pEC_50_ values for the WT, I212F, and M345R. (**D**) WT, I212F, and M345R maximal efficacy expressed as a percentage of the mean of the maximum of the WT same-day control (E_max_ (% of WT E_max_)). Statistical analysis was performed using one-way ANOVA with Dunnett’s post hoc test, or Student’s *t*-test as given in Table 2, and indicated as: * *p* < 0.05; ** *p* < 0.01; *** *p* < 0.001; ns—not significant; nd—not determined. Data shown as mean ± SEM.

In order to confirm the gain-of-function phenotype of I212F and M345R as reported [32,33,34,35], we performed the G-protein TRUPATH assay [40]. For this purpose, we used Gα_i/o_ subunits (Gα_i1_, Gα_i2_, Gα_i3_, Gα_oA_, and Gα_oB_) in combination with Gβ_3_ and Gγ_9_ or Gγ_8_ subunits, as recommended by Olsen et al. [40], and analyzed concentration–response curves with the native D2 ligand dopamine (Figure 2A).

In contrast to Rodriguez-Contreras et al., who used a combination of Gα together with β_1_ and γ_2_, we could not observe increased constitutive activities for I212F and M345R Gα_i2_ (please note that there is even a significant reduction for I212F Gα_i2_) and for I212F Gα_i3_ using Gα and β_3_ and γ_8_ or γ_9_ combinations. However, we could confirm increased constitutive activities for all other used subunit combinations (Figure 2B, Table 2). Further, we were not able to determine pEC_50_ for I212F, M345R Gα_oA_, and M345R Gα_oB_ due to high constitutive activities but could confirm the increased pEC_50_ for I212F and M345R Gα_i2_ and Gα_i3_. In addition, we could also identify an increased pEC_50_ for M345R Gα_i1_, but not for I212F Gα_i1_ and Gα_oB_ (Figure 2C; Table 2). Comparing the maximal response efficacy (E_max_) of the WT and the mutants, we could identify an increased E_max_ for I212F: Gα_oA_ and a trend in Gα_oB_. The E_max_ was unchanged for Gα_i1_ and α_i3_, and even reduced for Gα_i2_. Interestingly, M345R has an increased E_max_ for all Gα subunits except Gα_i2_ (Figure 2D; Table 2). Our results demonstrate that both mutations lead to a general G-protein signaling gain-of-function phenotype for most Gα-, β-, and γ-combinations, with a mixed phenotype for I212F Gα_i2_ (reduced basal activity, increased agonist sensitivity, and reduced E_max_). However, they also clearly indicate the phenotype dependency on the specific Gα, β, and γ subunit combinations used.

### 3.3. G-Protein Expression in Mouse and Human Brains

The G-protein subunits used in the experiments above were selected according to an optimized signal/noise ratio in the TRUPATH assay [40]. In order to identify which subunits are predominantly expressed together with the WT, we analyzed publicly available 10xRNA sequencing data from mice and humans, according to Yao et al. [42] and Siletti et al. [15].

**Figure 3:**
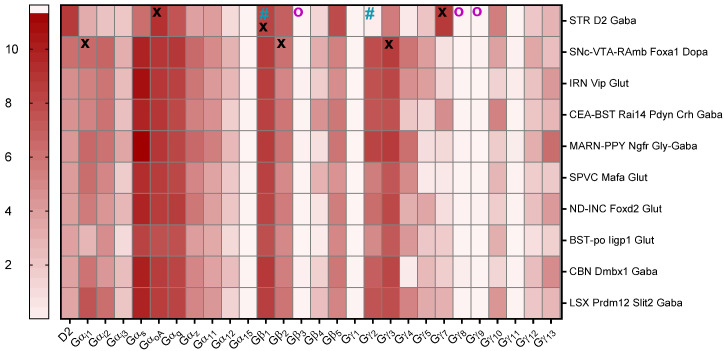
Heatmap of D2 (*Drd2*) and G-proteins RNA expression levels in different mouse brain regions/cell types. Data derived from the Allen Mouse Brain Cell Type Atlas [43]. The selected brain regions correspond to brain regions with the highest levels of *Drd2*. Data are plotted from white (lowest expression) to red (highest expression) as log2 copies per million (CPM) + 1 of 10xRNA (D2 = *Drd2*, Gα_i1_ = *Gna1*, Gα_i2_ = *Gna2*, Gα_i3_ = *Gna3*, Gα_s_ = *Gnas*, Gα_oA_ = *Gnao1*, Gα_q_ = *Gnaq*, Gα_s_ = *Gnas*, Gα_z_ = *Gnaz*, Gβ_1_ = *Gnb1*, Gβ_2_ = *Gnb2*, Gβ_3_ = *Gnb3*, Gβ_4_ = *Gnb4*, Gβ_5_ = *Gnb5*, Gγ_1_ = *Gngt1*, Gγ_2_ = *Gngt2*, Gγ_3_ = *Gng3*, Gγ_4_ = *Gng4*, Gγ_5_ = *Gng5*, Gγ_7_ = *Gng7*, Gγ_8_ = *Gng8*, Gγ_9_ = *Gng9*, Gγ_10_ = *Gng10*, Gγ_11_ = *Gng11*, Gγ_12_ = *Gng12*, Gγ_13_ = *Gn13*). The G-protein subunits used by Rodriguez-Contreras et al. are indicated by turquoise hash signs, while those used in our experiments above are marked with violet circles. Subunits with the highest levels are highlighted with a black X (please note that only Gα_i/o_ subunits have been included). The analyzed brain regions include: the corpus striatum (STR WT Gaba), the compact part of substantia nigra, the ventral tegmental area and midbrain raphe nuclei (SNc-VTA-Ramb Foxa1 Dopa), the intermediate reticular nucleus (IRN Vip Glut), the central amygdala nucleus and bed nuclei of the stria terminalis (CEA-BST Rai14 Pdyn Crh Gaba), the magnocellular reticular nucleus and parapyramidal nucleus (MARN-PPY Ngfr Gly-Gaba), the caudal part of the spinal nucleus of the trigeminal (SPVC Mafa Glut), the nucleus of Darkschewitsch and interstitial nucleus of cajal (ND-INC FoxWT Glut), bed nuclei of the stria terminalis and posterior complex of thalamus (BST-po ligp1p Glut), cerebellar nuclei (CBN Dmbx1 Gaba), and the lateral septal complex (LSX Prdm 12 Slit2 Gaba). For more details, please refer to Yao et al. [42].

**Figure 4:**
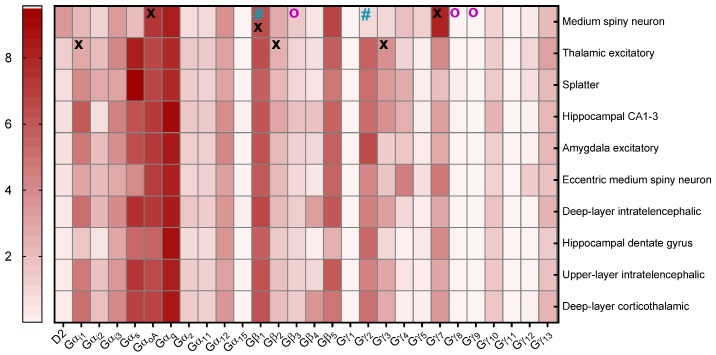
Heatmap of different D2 (Drd2) and G-proteins RNA expression levels across various human brain regions. The genetic datasets were extrapolated from the Allen Human Brain Cell Type Atlas [43]. The brain regions were selected based on the highest levels of Drd2. Values are plotted from white (lowest expression) to red (highest expression) as log2 copies per million (CPM) + 1 of 10xRNA (D2 = *Drd2*, Gα_i1_ = *Gna1*, Gα_i2_ = *Gna2*, Gα_i3_ = *Gna3*, Gα_s_ = *Gnas*, Gα_oA_ = *Gnao1*, Gα_q_ = *Gnaq*, Gα_s_ = *Gnas*, Gα_z_ = *Gnaz*, Gβ_1_ = *Gnb1*, Gβ_2_ = *Gnb2*, Gβ_3_ = *Gnb3*, Gβ_4_ = *Gnb4*, Gβ_5_ = *Gnb5*, Gγ_1_ = *Gngt1*, Gγ_2_ = *Gngt2*, Gγ_3_ = *Gng3*, Gγ_4_ = *Gng4*, Gγ_5_ = *Gng5*, Gγ_7_ = *Gng7*, Gγ_8_ = *Gng8*, Gγ_9_ = *Gng9*, Gγ_10_ = *Gng10*, Gγ_11_ = *Gng11*, Gγ_12_ = *Gng12*, Gγ_13_ = *Gn13*). The subunits used by Rodriguez-Contreras et al. are indicated by turquoise hash signs, while those used in our experiments above are marked with violet circles. Subunits with the highest levels are highlighted with a black X (please note that only Gα_i/o_ subunits have been included). For more details, please refer to Siletti et al. [15].

An examination of the expression levels of different G-protein subunits in specific cell types/brain regions across mice (Figure 3) and humans (Figure 4) revealed that, in both, Gα_oA_ is the most highly expressed Gα subunit followed by Gα_i1_, while Gβ_1_ and β_2_, as well as Gγ_3_ and γ_7_, are the predominant Gβ and Gγ subunits, respectively. We therefore concluded that the I212F and M345R G-protein signaling phenotypes should be investigated using these subunit combinations.

### 3.4. Effect of I212F and M345R on Gα_oA_ and α_i1_ in Combination with β_1_ and β_2_ and γ_3_ and γ_7_

**Figure 5:**
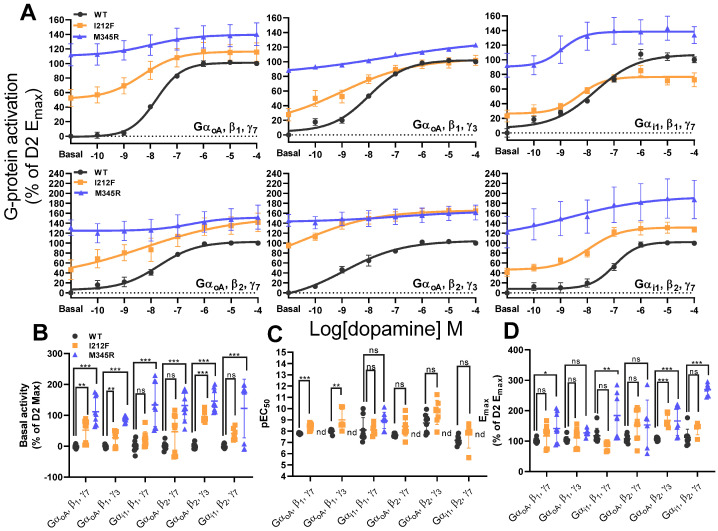
Most abundant expressed G-protein combination activation of the WT, I212F, and M345R. (**A**) Concentration–response curves for the WT, I212F, and M345R with Gα_i/o_, β-, and γ-subunits as indicated, following dopamine application. netBRET (GFP2/Rluc8) values are normalized to the mean of the max dopamine response of the WT same-day control and reported as a percentage of the WT same-day control (% of G-protein activation (WT E_max_)). (**B**) Basal activity for the WT, I212F, and M345R are plotted as percentages of the mean of the WT E_max_ same-day control (Basal activity (% of WT Max)). (**C**) Comparison of pEC_50_ values for the WT, I212F, and M345R. (**D**) WT, I212F, and M345R maximal efficacy expressed as a percentage of the mean maximum of the WT same-day control (E_max_ (% of WT E_max_)). Statistical analysis was performed using one-way ANOVA with Dunnett’s post hoc test, or Student’s *t*-test as given in Table 3, and indicated as: * *p* < 0.05; ** *p* < 0.01; *** *p* < 0.001; ns—not significant; nd—not determined. Data shown as mean ± SEM.

In order to investigate the effect of I212F and M345R on the specific Gα-, β-, and γ-subunit combinations, we performed concentration–response experiments as described above (Figure 5A).

Using the specific subunit combinations, we could confirm the increased constitutive activities of I212F and M345R Gα with all tested β and γ combinations and a trend for I212F Gα_oA_, β_2_, and γ_7_, excluding I212F Gα_i1_, β_1_ and γ_7_, and β_2_ and γ_7_ (Figure 5B, Table 3). In contrast to the experiments for I212F above, and consistent with Rodriguez-Contreras et al., we could detect a significantly increased I212F dopamine pEC_50_ for Gα_oA_, β_1_ and, γ_7_, and β_1_ and γ_3_, as well as a trend for β_2_ and γ_3_, but not for Gα_oA_ and α_i1_ in combination with β_2_ and γ_7_, and Gα_i1_ β_1_ and γ_7_. Interestingly, we could not estimate pEC_50_ values for most Gα–, β–, and γ- M345R combinations, due to high constitutive activities and the lack of a sufficient dopamine response. Additionally, we could detect a trend for an increased M345R pEC_50_ for Gα_i1_, β_1_, and γ_7_, consistent with Rodriguez-Contreras et al. and our experiments above (Figure 5C; Table 3). M345R E_max_ was increased for all tested subunit combinations, excluding Gα_oA_, β_2_, and γ_7_ and a trend in Gα_oA_, β_1_, and γ_3_. In contrast to M345R, for I212F, we could only detect a significantly increased E_max_ for Gα_oA_, β_2_, and γ_3_ and a trend for Gα_i1_, β_2_, and γ_7_ (Figure 5D, Table 3).

## 4. Conclusions

In recent years, heterozygous variants in dopamine WT receptors have been discovered as a cause of chorea and dystonia [33,34]. In this study, we investigate the G-protein- and β-arrestin-mediated signaling pathways of the WT receptor mutations I212F and M345R, and compare the results with Rodriguez-Contreras et al. [32,33,34,35]. The use of the PRESTO-Tango assay to investigate β-arrestin 2 recruitment allowed us to provide data on the receptor’s constitutive activities in the WT and mutants. We could show that constitutive activity is significantly increased for M345R, but not for I212F. In contrast to Rodriguez-Contreras et al., our findings on β-arrestin 2 recruitment reveal a significant reduction in E_max_ for M345R, while it remained unchanged for I212F. This effect could be explained by either the increase in M345R constitutive activity or the use of different assays, ligands, or GRK expression [44,45,46]. We are therefore able to extend the existing knowledge on the molecular phenotype of mutant M345R by increased basal β-arrestin 2 recruitment, which has to be considered as a mixed phenotype, leading to a gain-of-function for β-arrestin 2 signaling, but a loss of function for mutant G-protein interactions [24]. However, we also have to mention that in comparison to Rodriguez-Contreras et al., we were not able to use dopamine for the PRESTO-Tango assay due to technical limitations.

Consistent with previous studies [32], we demonstrate that D2 receptors, in the presence of their native ligand dopamine, can signal via different heterotrimeric G-protein combinations in vitro, using heterologous overexpression systems [47,48,49]. For this purpose, we employed a BRET assay, which is dependent on the dissociation of the Gα subunit fused to RLuc8 from the βγ dimer (γ fused to GFP2), which allowed us to investigate G-protein recruitment for both wild-type and mutated receptors [40]. In contrast to Rodriguez-Contreras et al. [32], we also report the maximal efficacy of heterotrimeric G-protein complex recruitment of the WT and mutants and can also report a mixed G-protein-dependent phenotype including gain-of-function (I212F: Gα_oA_ and a trend in Gα_oB_; M345R: Gα_i1_, α_i2_, α_oA_, and α_oB_), unchanged (I212F: Gα_i1_ and α_i3_; M345R: Gα_i2_), and, importantly, even loss-of-function (I212F: Gα_i2_; decreased basal activity and E_max_). Although we could confirm most of Rodriguez-Contreras et al.’s reported findings in constitutive activities and ligand sensitivities, we could still detect significant differences, including decreased constitutive activity for I212F Gα_i2_, which was unchanged for M345R and no difference was found for I212F ligand potency for Gα_i1_. The observed differences in mutant phenotype can potentially be explained by the alternate heterotrimeric G-protein combination used (Gα_i2_, β_3_, γ_9_). Therefore, it is very important to define the most accurate G-protein combination, if possible. In addition, a subunit-specific bias in heterotrimeric G-protein signaling has recently been reported for D2 ligands [50], which could explain the observed differences. The different D2 ligands used may also lead to a G-protein subunit bias. We therefore conclude that for G-protein activation sensitivity and efficacy, the natural ligand dopamine should be used to gain a more complete understanding of receptor function. The discrepancies between Rodriguez-Contreras et al.’s results and our findings clearly demonstrate that specific G-protein signaling is not only dependent on Gα but also on the combination of β- and γ-subunits [51,52,53]. We therefore wanted to investigate the WT and mutants using specific G-protein complexes, which are highly co-expressed in different brain regions or cell types responsible for motor coordination.

For this purpose, we took advantage of the publicly available data in the Allen Brain Cell Type Atlas [43], which allowed us to detect the expression levels of different G-protein subunits in different cell types/brain regions in both mice [42] and humans [15]. This data revealed that neither the β_1_ and γ_2_ combination used by Rodriguez-Contreras et al. [32], nor the β_3_ and γ_8_ or γ_9_ combinations used by us initially, represent the predominantly expressed heterotrimeric G-protein subunit combinations in basal ganglia. We therefore repeated the experiments above using the more relevant Gα_oA_ or Gα_i1_, β_1_ or β_2_, and γ_3_ or γ_7_ combinations. In contrast to published data [32,35], we were not able to estimate the pEC_50_ for most tested Gα-, β-, and γ-M345R combinations (excluding Gα_i1_, β_1_, γ_7_), as the lack of a dopamine response and the increased constitutive activity did not allow us to fit individual concentration–response curves, representing a gain-of-function phenotype which exceeded previously reported levels [32]. Our data could however confirm increased constitutive activity of I212F and M345R Gα_oA_ with all tested β and γ combinations, except for I212F with Gα_i1_, β_1_, γ_7_, and Gα_i1_ or α_oA_ with β_2_ and γ_7_. This is in line with the results reported by Rodriguez-Contreras et al., who also observed increased constitutive activity using the combination of Gα_oA_ and α_i1_ with β_1_ and γ_2_. However, our experiments clearly indicate that the β- and γ-subunit combinations have a substantial influence on WT and mutant G-protein signaling. In their study, Rodriguez-Contreras et al. investigated both the long and short isoforms of the WT fused to an optimized version of RLuc8 and mVenus-tagged β-arrestin 2, allowing for BRET measurement. Here, we quantify β-arrestin 2 recruitment with the PRESTO-Tango assay, which depends on β-arrestin-2 fused to the TEV protease, as well as a luciferase reporter gene, which are both stably expressed in the HTLA cell line. Following β-arrestin 2 recruitment, GPCR C-terminally fused tTA is released after TEV side cleavage, dislocated to the nucleus, and followed by reporter gene expression. For measuring G-protein activation, Rodriguez-Contreras et al. fused RLuc8 to different Gα subunits, and mVenus fragments V1 and V2 were fused to Gβ_1_ and Gγ_2_, respectively. In contrast, the TRUPATH assay reports the dislocation of RLuc8-tagged Gα proteins and GFP2-tagged Gγ proteins. Although we could confirm the general gain-of-function phenotype for both mutants, I212F and M345R, we cannot exclude that the observed differences are a result of the alternative assays used.

In general, we are able to confirm the gain-of-function phenotype with the G-protein subunit combinations highly expressed in brain regions involved in motor coordination, with a more prominent gain-of-function phenotype for M345R. This is in line with the more severe phenotype reported for the two patients carrying the M354R mutation, with the onset of chorea within the first year of life and additional features, which were not described in patients with the I212F mutation, such as cognitive impairments, developmental delays, and behavioral problems [33]. Within this study, we aim to provide a more detailed evaluation of functional G-protein signaling consequences of the WT variants, I212F and M345R, underlying chorea movement disorders. Although we could detect substantial differences to published data using different G-protein transducers [40], we could generally confirm the gain-of-function phenotype of WT I212F and M345R mutants. Our data clearly demonstrate that the co-expression of different G-protein subunits affects the molecular disease phenotype, even as drastically as eliciting an apparent loss-of-function in WT signaling as in the case for I212F and Gα_i2_; however, this is not expressed in the relevant cell type at significant levels. Therefore, publicly available expression level data should be implemented in studies investigating the effect of mutations on G-protein couplings, prioritizing the most abundantly expressed G-protein combinations.

## Figures and Tables

**Table 1:** β-arrestin 2 recruitment for the WT, I212F, and M345R. pEC_50_, basal activities, and E_max_ are indicated. Data are shown as mean ± SEM. Statistical analysis was conducted using one-way ANOVA with Dunnett’s post hoc test.

β-arrestin 2	WT	I212F		M345R		Anova	n/N
**pEC_50_**	9.08 ± 0.10	8.17 ± 0.15	*p* = 0.036	6.56 ± 0.57	*p* < 0.001	F(2,45) = 22.99, *p* < 0.001	9/3
**Basal activity**	2.21 ± 1.02	0.64 ± 1.21	*p* = 0.661	14.60 ±1.89	*p* < 0.001	F(2,31) = 28.40, *p* < 0.001	9/3
**E_max_**	86.60 ± 2.47	78.32 ± 3.75	*p* = 0.134	51.97 ± 3.96	*p* < 0.001	F(2,44) = 29.99, *p* < 0.001	9/3

**Table 2:** G-protein signaling of the WT, I212F, and M345R. pEC_50_, Basal activity, and E_max_ are shown. Values are shown as mean ± SEM. Statistical analysis was performed using one-way ANOVA with Dunnett’s post hoc test, or Student’s *t*-test, as indicated. nd—not determined.

pEC_50_											
	Gα_i1_, β_3_, γ_9_		Gα_i2_, β_3_, γ_9_		Gα_i3_, β_3_, γ_9_		Gα_oA_, β_3_, γ_8_		Gα_oB_, β_3_, γ_8_		n/N
WT	6.95 ± 0.04		7.72 ± 0.09		6.90 ± 0.02		0.18 ± 0.21		7.38 ± 0.08		9/3
I212F	7.27 ± 0.08	*p* = 0.122	8.76 ± 0.41	*p* = 0.010	7.63 ± 0.07	*p* < 0.001	nd	-	7.93 ± 0.31	*p* = 0.110	9/3
M345R	7.80 ± 0.18	*p* < 0.001	9.03 ± 0.11	*p* = 0.002	9.13 ± 0.18	*p* < 0.001	nd	-	nd	-	9/3
Anova	F(2,24) = 13.13, *p* < 0.001		F(2,19) = 8.70, *p* = 0.002		F(2,19) = 161.40, *p* < 0.001		-		*t*-test		
**Basal activity**											
	**Gα_i1_, β_3_, γ_9_**		**Gα_i2_, β_3_, γ_9_**		**Gα_i3_, β_3_, γ_9_**		**Gα_oA_, β_3_, γ_8_**		**Gα_oB_, β_3_, γ_8_**		**n/N**
WT	0.00 ± 3.16		0.00 ± 20.60		0.00 ± 1.53		0.00 ± 2.01		0.00 ± 2.89		9/3
I212F	43.99 ± 2.65	*p* < 0.001	−26.09 ± 8.27	*p* = 0.023	−11.57 ± 7.46	*p* = 0.639	102.20 ± 7.99	*p* < 0.001	64.05 ± 13.81	*p* = 0.002	9/3
M345R	83.35 ± 3.50	*p* < 0.001	20.60 ± 7.04	*p* = 0.111	46.66 ± 15.85	*p* = 0.007	115.50 ± 5.66	*p* < 0.001	116.60 ± 14.66	*p* < 0.001	9/3
Anova	F(2,24) = 178.20, *p* < 0.001		F(2,21) = 10.43, *p* < 0.001		F(2,24) = 9.22, *p* = 0.001		F(2,24) = 119.99, *p* < 0.001		F(2,24) = 24.72, *p* < 0.001		
**E_max_**											
	**Gα_i1_, β_3_, γ_9_**		**Gα_i2_, β_3_, γ_9_**		**Gα_i3_, β_3_, γ_9_**		**Gα_oA_, β_3_, γ_8_**		**Gα_oB_, β_3_, γ_8_**		**n/N**
WT	98.53 ± 2.42		100.90 ± 1.66		102.56 ± 0.88		102.93 ± 1.97		103.32 ± 1.70		9/3
I212F	107.67 ± 3.68	*p* = 0.384	42.42 ± 3.27	*p* < 0.001	102.25 ± 3.28	*p* = 0.997	155.86 ± 2.41	*p* < 0.001	132.32 ± 13.54	*p* = 0.055	9/3
M345R	144.54 ± 8.07	*p* < 0.001	105.79 ± 6.79	*p* = 0.591	125.18 ± 6.23	*p* < 0.001	172.24 ± 3.84	*p* < 0.001	175.62 ± 4.66	*p* < 0.001	9/3
Anova	F(2,24) = 21.06, *p* < 0.001		F(2,18) = 74.34, *p* < 0.001		F(2,21) = 12.31, *p* < 0.001		F(2,23) = 161.90, *p* < 0.001		F(2,20) = 12.18, *p* < 0.001		

**Table 3:** Most abundant expressed G-protein combination signaling for the WT, I212F, and M345R. pEC_50_, Basal activity, and E_max_ are given. Values are shown as mean ± SEM; nd—not determined. Statistical analysis was performed using one-way ANOVA with Dunnett’s post hoc test, or Student’s *t*-test, as indicated. nd—not determined.

pEC_50_													
	Gα_oA_, β_1_, γ_7_		Gα_oA_, β_1_, γ_3_		Gα_i1_, β_1_, γ_7_		Gα_oA_, β_2_, γ_7_		Gα_oA_, β_2_, γ_3_		Gα_i1_, β_2_, γ_7_		n/N
WT	7.80 ± 0.02		7.99 ± 0.06		8.10 ± 0.38		7.64 ± 0.06		8.72 ± 0.26		7.18 ± 0.14		9/3
I212F	8.40 ± 0.09	*p* < 0.001	9.00 ± 0.38	*p* = 0.011	8.09 ± 0.18	*p* = 0.999	8.42 ± 0.29	*p* = 0.050	9.65 ± 0.41	*p* = 0.415	7.67 ± 0.52	*p* = 0.289	9/3
M345R	nd		nd		8.95 ± 0.18	*p* = 0.091	nd		nd		nd		9/3
Anova	*t*-test		*t*-test		F(2,20) = 2.84, *p* = 0.082		*t*-test		*t*-test		*t*-test		
**Basal activity**													
	**Gα_oA_, β_1_, γ_7_**		**Gα_oA_, β_1_, γ_3_**		**Gα_i1_, β_1_, γ_7_**		**Gα_oA_, β_2_, γ_7_**		**Gα_oA_, β_2_, γ_3_**		**Gα_i1_, β_2_, γ_7_**		**n/N**
WT	0.00 ± 1.86		0.00 ± 1.71		0.00 ± 5.80		0.00 ± 4.08		0.00 ± 2.89		0.00 ± 2.23		9/3
I212F	52.37 ± 11.81	*p* = 0.005	27.64 ± 8.48	*p* = 0.002	23.08 ± 8.07	*p* = 0.440	46.82 ± 19.93	*p* = 0.062	95.13 ± 5.33	*p* < 0.001	41.62 ± 7.92	*p* = 0.334	9/3
M345R	111.87 ± 15.14	*p* < 0.001	88.30 ± 3.16	*p* < 0.001	133.67 ± 23.27	*p* < 0.001	131.34 ± 15.34	*p* < 0.001	145.76 ± 11.83	*p* < 0.001	122.23 ± 31.67	*p* < 0.001	9/3
Anova	F(2,24) = 25.26, *p* < 0.001		F(2,24) = 72.21, *p* < 0.001		F(2,24) = 23.91, *p* < 0.001		F(2,24) = 20.48, *p* < 0.001		F(2,24) = 93.01, *p* < 0.001		F(2,21) = 9.72, *p* < 0.001		
**E_max_**													
	**Gα_oA_, β_1_, γ_7_**		**Gα_oA_, β_1_, γ_3_**		**Gα_i1_, β_1_, γ_7_**		**Gα_oA_, β_2_, γ_7_**		**Gα_oA_, β_2_, γ_3_**		**Gα_i1_, β_2_, γ_7_**		**n/N**
WT	101.38 ± 2.46		105.43 ± 3.25		117.75 ± 7.99		107.36 ± 4.07		107.50 ± 1.82		112.01 ± 8.96		9/3
I212F	117.21 ± 11.89	*p* = 0.532	111.57 ± 10.26	*p* = 0.753	83.23 ± 4.85	*p* = 0.194	152.83 ± 16.34	*p* = 0.125	168.10 ± 6.70	*p* < 0.001	141.98 ± 9.34	*p* = 0.068	9/3
M345R	141.65 ± 15.92	*p* = 0.039	127.29 ± 4.23	*p* = 0.081	184.23 ± 24.78	*p* = 0.008	153.16 ± 33.17	*p* = 0.176	166.43 ± 18.07	*p* < 0.001	269.70 ± 8.12	*p* < 0.001	9/3
Anova	F(2,21) = 3.08, *p* = 0.065		F(2,22) = 2.36, *p* = 0.118		F(2,22) = 11.54, *p* < 0.001		F(2,21) = 2.30, *p* = 0.125		F(2,22) = 13.22, *p* < 0.001		F(2,17) = 83.76, *p* < 0.001		

## Data Availability

All data are available from the authors upon reasonable request.
